# Prognostic role of the *CDNK1B* V109G polymorphism in multiple endocrine neoplasia type 1

**DOI:** 10.1111/jcmm.12552

**Published:** 2015-03-30

**Authors:** Luisa Circelli, Valeria Ramundo, Vincenzo Marotta, Concetta Sciammarella, Francesca Marciello, Michela Del Prete, Lina Sabatino, Daniela Pasquali, Francesco Izzo, Stefania Scala, Annamaria Colao, Antongiulio Faggiano, Vittorio Colantuoni

**Affiliations:** a Oncological Immunology, Department of Abdominal Oncology, National Cancer Institute, “Fondazione G. Pascale”Naples, Italy; b CEINGE Advanced BiotechnologiesNaples, Italy; c Department of Clinical Medicine and Surgery, “Federico II” University of NaplesNaples, Italy; d Department of Sciences and Technologies, University of SannioBenevento, Italy; e Department of Cardiothoracic and Respiratory Sciences, Endocrinology Unit, Second University of NaplesNaples, Italy

**Keywords:** *CDKN1B*, polymorphisms, MEN1, neuroendocrine tumours, prognosis

## Abstract

*CDKN1B* encodes the cyclin-dependent kinase inhibitor p27/Kip1. *CDKN1B* mutations and polymorphisms are involved in tumorigenesis; specifically, the V109G single nucleotide polymorphism has been linked to different tumours with controversial results. Multiple endocrine neoplasia type 1 (MEN1) is a rare autosomal dominant syndrome, characterized by the development of different types of neuroendocrine tumours and increased incidence of other malignancies. A clear genotype–phenotype correlation in MEN1 has not been established yet. In this study, we assessed whether the *CDKN1B* V109G polymorphism was associated with the development of aggressive tumours in 55 consecutive patients affected by MEN1. The polymorphism was investigated by PCR amplification of germline DNA followed by direct sequencing. Baseline and follow-up data of tumour types and their severity were collected and associated with the genetic data. MEN1-related aggressive and other malignant tumours of any origin were detected in 16.1% of wild-type and 33.3% of polymorphism allele-bearing patients (*P* = NS). The time interval between birth and the first aggressive tumour was significantly shorter in patients with the *CDKN1B* V109G polymorphism (median 46 years) than in those without (median not reached; *P* = 0.03). Similarly, shorter was the time interval between MEN1 diagnosis and age of the first aggressive tumour (*P* = 0.02). Overall survival could not be estimated as 96% patients were still alive at the time of the study. In conclusion, *CDKN1B* V109G polymorphism seems to play a role in the development of aggressive tumours in MEN1.

## Introduction

Multiple endocrine neoplasia type 1 (MEN1) is an inherited tumour syndrome characterized by the occurrence of tumours of the parathyroid glands, anterior pituitary, pancreatic islets and the adrenal glands, as well neuroendocrine tumours (NETs) of the foregut (thymic, bronchial and gastric carcinoids). Other non-endocrine tumours of the skin (angiofibroma, lipoma, collagenoma) and central nervous system (ependimoma, meningioma) may be associated 1. According to the MEN consensus panel, the clinical diagnosis of this syndrome is based on the concomitant occurrence of at least two of the three MEN1-related endocrine tumours (parathyroid adenoma, pituitary adenoma, pancreatic NET). Familial MEN1 is defined by the presence of at least one MEN1-related NET plus at least one-first-degree relative with just one of the three classical tumours or a known *MEN1* germline mutation 2. MEN1 is, in fact, caused by mutations of the *MEN1* gene mapped on chromosome 11q13 3. More than 1336 different mutations (1133 germline and 203 somatic) have been reported 4, the majority of which are inactivating according to the notion that *MEN1* is a tumour suppressor gene. No *MEN1* mutational hot spots have been detected and, up to date, a clear correlation between genetic events and the variable clinical expression of the disease has not been established 4,5. The gene product, menin, is an adaptor protein that interacts with multiple partners involved in several cellular processes, including transcription regulation, DNA replication and repair and signal transduction. It is also essential for viability, as reported in *Men1* null mutant mice 6. Menin targets several genes including homeobox domain (HOX), human telomerase (hTERT) and nuclear receptor genes and *CDKN2C* and *CDKN1B* 7–10. Specifically, *CDKN1B* encodes a regulatory protein that controls the progression from the G1 to the S phase of the cell cycle. Loss-of-function mutations of the gene have been described and contribute to tumorigenesis, in agreement with the notion that aberrant cell cycle control is one of the hallmarks of cancer 11,12. *Cdkn1b* mutations in rat are linked to the development of the MENX syndrome characterized by a clinical picture that overlaps MEN1 and MEN2 syndromes. Consistently, *CDKN1B* mutations are responsible for the MEN4 syndrome in humans that displays the same features as MENX 13. In addition to gene mutations, at least 21 gene polymorphisms have been reported and variably associated with tumour progression. Studies have mainly been focused on a single nucleotide polymorphism (SNP; database: rs2066827) that replaces a valine (V) with a glycine (G) residue at codon 109 and on its relationship with cancer risk. Controversial results have been reported: two studies showed that the G polymorphic allele is associated with an increased risk of oral squamous cell carcinoma and prostate cancer 14,15 whereas others suggested an association with a decreased risk of breast cancer 16 or a more favourable disease progression in sporadic medullary thyroid carcinoma 17. Other reports, finally, showed no association of the *CDKN1B* V109G polymorphism with breast 18,19 pancreatic 20,21 and prostate cancer risk 22.

The aim of this study was to verify whether the *CDKN1B* V109G polymorphism is linked to aggressive tumours in MEN1 patients.

## Materials and methods

### Patients

Fifty-five consecutive MEN1 patients (22 males and 33 females, age range 5–82 years, belonging to 20 families), followed at the Neuroendocrine Tumours Unit, Department of Clinical Medicine and Surgery of the ‘Federico II’ University of Naples, were prospectively enrolled in this study starting from 2005. Patients’ characteristics are reported in Table1. All patients had a diagnosis of MEN1 according to guidelines 2,23: 52 of them were positive and three negative at the genetic screening for *MEN1* mutations 2. Twenty of 55 patients had clinically evident MEN1 manifestations, whereas 35 were their first-degree relatives, identified as gene mutations carriers through the genetic screening. Patients were screened for MEN1-related aggressive and other malignant tumours of any origin. MEN1-related aggressive tumours were defined as follows: (*i*) a pancreatic NET >2 cm in size 23, and (*ii*) a thoracic (bronchial or thymic) NET of any stage 23. Ninety cases, age- and gender-matched with the enrolled patients, served as control. Thirty-six male and fifty-four female individuals with no history of tumours were recruited among the medical and paramedical personnel of the institutions participating in this study. These criteria were designed to ensure that all of them had a minimal risk of having or ever developing MEN1. After informed consent was obtained, each participant was interviewed using a pretested questionnaire to obtain information on medical history, lifestyles and family history of cancer up to first-degree relatives. Based on this information, the control group does not include individuals with history of breast, hereditary prostate and pancreatic tumours, or any other cancer. All patients and controls were of European descent with a nationwide distribution. Tumour type, clinical manifestations and outcome data collected for all patients were related to the *CDNK1B* genetic profile. All MEN1 patients enrolled in this study received the same clinical, laboratory and imaging follow-up, according to the most recent MEN1 guidelines 23.

**Table 1:** Patients’ characteristics

Family	Patient no.	Sex	Age at *CDKN1B* analysis (years)	*MEN1* mutation (NM.130803.2)	*CDKN1B*	MEN1-related manifestations	Age at diagnosis of the first MEN1 manifestation
1	1	F	50	c.303delC, exon 2	POL	PAH, PA, pNET	42
1	2	F	25	c.303delC, exon 2	POL	PAH, pNET	18
1	3	M	52	c.303delC, exon 2	WT	PAH, PA, pNET	44
1	4	M	26	c.303delC, exon 2	WT	PAH, PA	20
1	5	F	23	c.303delC, exon 2	WT	PA	16
2	6	M	41	c.1046delC, exon 7	POL	PAH, pNET	32
2	7	F	56	c.1046delC, exon 7	POL	PAH, PA, pNET, AT	47
2	8	M	35	c.1046delC, exon 7	POL	PAH, pNET	29
3	9	F	49	c.673T>A, p.W225R, exon 4	POL	PAH	32
3	10	F	26	c.673T>A, p.W225R, exon 4	POL	PAH, PA, pNET	18
4	11	M	34	c.451delAAG, exon 2	POL	pNET, tNET	32
4	12	M	62	c.451delAAG, exon 2	WT	PAH, PA, pNET, AT	51
4	13	F	38	c.451delAAG, exon 2	WT	PAH, pNET	36
4	14	F	46	c.451delAAG, exon 2	WT	PAH, pNET, AT	42
4	15	M	12	c.451delAAG, exon 2	WT	–	–
4	16	F	15	c.451delAAG, exon 2	WT	–	–
4	17	M	38	c.451delAAG, exon 2	WT	PAH	36
4	18	F	28	c.451delAAG, exon 2	WT	PAH, PA	26
4	19	F	59	c.451delAAG, exon 2	WT	PAH, pNET	57
4	20	M	58	c.451delAAG, exon 2	WT	PAH, pNET, AT	57
5	21	M	49	c.335delA, exon 2	WT	PAH, PA, pNET, AT	39
6	22	M	53	c.502G>A,p.G168R exon 3	POL	PAH, PA, pNET, AT	49
7	23	F	36	c.557C>A, p.H186R exon3	POL	PAH, PA, pNET	33
7	24	M	16	c.557C>A, p.H186R exon3	POL	–	–
7	25	M	6	c.557C>A, p.H186R exon3	POL	–	–
7	26	F	53	c.557C>A, p.H186R exon3	POL	pNET	53
7	27	M	62	c.557C>A, p.H186R exon3	POL	PAH	61
7	28	M	60	c.557C>A, p.H186R exon3	POL	PAH, PA	59
7	29	M	66	c.557C>A, p.H186R exon3	WT	PAH	45
8	30	M	51	c.825+1G>A intr5	POL	PAH, PA, pNET, AT	49
8	31	M	20	c.825+1G>A intr5	POL	PAH, PA, pNET	19
9	32	F	43	c.95C>G, p.P32R exon2	POL	PAH, PA, tNET	29
9	33	F	82	c.95C>G, p..P32R exon2	WT	–	81
10	34	F	60	c.1576del11(delACTGTCGCTGG), exon 10	WT	PAH, PA, pNET	52
10	35	F	30	c.1576del11(delACTGTCGCTGG), exon 10	WT	PAH, PA, pNET	17
11	36	M	35	c.1576del11(delACTGTCGCTGG), exon 10	WT	PAH, PA, pNET	30
11	37	F	62	c.1576del11(delACTGTCGCTGG), exon 10	WT	PAH, PA, pNET	57
12	38	M	42	c.1065+1G>A, intron 7	WT	PAH, PA, pNET	37
13	39	F	32	c.799-9G>A, intron 4	WT	PAH, PA, pNET	27
13	40	M	31	c.799-9G>A, intron 4	WT	pNET	28
13	41	F	51	c.799-9G>A, intron 4	WT	PAH, pNET, AT	48
13	42	F	8	c.799-9G>A, intron 4	WT	–	–
14	43	F	53	c.518T>C, p.L173P, exon 3	WT	PAH, pNET	36
14	44	F	30	c.518T>C, p.L173P, exon 3	WT	PAH, pNET	28
14	45	F	49	c.518T>C, p.L173P, exon 3	WT	PAH, pNET	47
14	46	F	24	c.518T>C, p.L173P, exon 3	WT	PAH	24
15	47	F	50	c.1061del C, exon 7	WT	PAH, pNET	40
16	48	M	42	c.1339C>T, p.Q447X, exon 9	WT	PAH, PA, pNET, AT	41
17	49	F	39	c.1258C>T, p.R420X, exon 9	WT	PAH, PA, pNET	38
17	50	F	5	c.1258C>T, p.R420X, exon 9	POL	–	–
17	51	F	19	c.1258C>T, p.R420X, exon 9	POL	–	–
17	52	F	12	c.1258C>T, p.R420X, exon 9	POL	–	–
18	53	F	58	Negative	POL	PAH, PA, pNET, renal carcinoma	45
19	54	F	34	Negative	POL	PAH, pNET	25
20	55	F	44	Negative	POL	PAH, PA	43

PAH: parathyroid adenoma/hyperplasia; PA: pituitary adenoma; pNET: pancreatic neuroendocrine tumour; tNET: thoracic neuroendocrine tumour; AT: adrenal tumour.

### DNA sequencing

Patients were tested for the presence of *MEN1* point mutations on the germline DNA extracted from peripheral blood. *MEN1* exons 2–10 were PCR amplified and automatically sequenced as previously described 24. Patients tested negative were subjected to the multiplex ligation-dependent probe amplification (MLPA) technique using the *MEN1* MLPA kit (MRC Holland, Amsterdam, The Netherlands) to identify and evaluate possible *MEN1* large deletions. Patients and controls were also tested for the *CDKN1B* V109G polymorphism on germline DNA by PCR amplification of exon 1 followed by direct sequencing using the primers and conditions previously described 17. Indeed, both *CDKN1B* exons 1 and 2 were screened for the detection of any other genetic variations in this gene, as reported 17. Written informed consent for all genetic screenings, blood samples handling and processing and other clinical procedures was provided by all investigated participants in accordance with the guidelines approved by the local ethical committee and with the Helsinki declaration.

### Statistical analysis

The statistical analysis was performed by SPSS for Windows version 20.0 (SPSS, Inc., Chicago, IL, USA). Data are reported as mean ± SEM. The significance was set at 5% (*P* < 0.05). Comparison between the numerical data was performed by the anova test and Student’s *t*-test was used to assess differences between two groups. The comparison between the categorical data was performed by the chi-squared test with the Yates correction, Fisher exact test or McNemar test as appropriate. The time interval between date of birth and appearance of the first aggressive tumour as well as that between MEN1 diagnosis and first aggressive tumour was calculated according to the Kaplan–Meier method and log rank test 25.

## Results

### Clinical and genetic findings

Taking into account the main MEN1-related manifestations, 42 patients (76%) had primary hyperparathyroidism, 23 (42%) pituitary adenoma, 33 (60%) pancreatic NETs, 2 (4%) thoracic NET and 7 (13%) adrenocortical tumour (Table1). The entire *CDKN1B* gene was investigated testing 55 patients and 90 matched controls. No mutations or other polymorphisms were detected along the entire gene (exons 1 and 2) with the exception of the T/G transversion at nucleotide 326 of exon 1 (rs2066827) that causes a valine (GTC) for glycine (GGC) substitution at position 109 of the mature protein. A significant difference in the frequency of the wild-type (T/T, 56.4%) and polymorphic alleles (T/G 38.2%, G/G 5.4%) was found between patients and controls (*P* = 0.001, 95% CI = 0.287–0.076; Table2). The frequency of the wild-type (T/T 56%) *versus* the polymorphic alleles (T/G and G/G 44%) among our MEN1 patients was not different from that reported for other types of tumours 15,16. The *CDKN1B* V109G polymorphism was detected in 24 of 55 MEN1 patients (44%). On this basis, patients were divided into two groups: group A (24 patients, 10M and 14F, bearing the polymorphism) and group B (31 patients, 12M and 19F, bearing the wild-type allele).

**Table 2:** Relative frequency of the T/G (V109G) polymorphism in MEN1 patients and control individuals

	*CDKN1B* Genotype		
DNA	T/T	T/G	G/G
Protein	V109V	V109G	G109G
Cases (*n* = 55)	31 (56.4%)	21 (38.2%)	3 (5.4%)
Controls (*n* = 90)	56 (62.2%)	28 (31.1%)	6 (6.7%)

No differences between group A and B were detected taking into account either gender or diagnostic circumstances (clinical diagnosis *versus* genetic screening; *P* = NS). A strong association was, instead, found with the *MEN1* genotype (*P* = 0.0001); in particular, three types of *MEN1* mutations [c.502G>A, p.G168R in exon 3; c.673T>A, p.W225R in exon 4; c.825+1G>A in intron 5] were only found in group A, that included also the patients tested negative for *MEN1* mutations. In contrast, six *MEN1* mutations [c.335delA in exon 2; c.518T>C, p.L173P in exon 3; c.1339C>T, p.Q447X in exon 9; c.1576del11 (delACTGTCGCTGG) in exon 10; c.799-9G>A in intron 4; c.1065+1G>A in intron 7] were only found in group B.

### Genotype/phenotype correlation

No significant differences in the frequency of the typical MEN1-related manifestations (primary hyperparathyroidism, pituitary adenoma, pancreatic NET) were detected between the two groups. At the time of the study, 6/24 (25%) patients of group A and 4/31 (13%) of group B had not yet clinical or radiological evidence of MEN1-related manifestations (*P* = NS). The age at diagnosis of the first MEN1-related manifestation was similar in group A (37.6 ± 3.1 years, range 18–61) and in group B (39.3 ± 2.7 years, range 16–81; *P* = NS). In contrast, the age at diagnosis of the first aggressive tumour was lower in group A (35.2 ± 3.9 years, range 19–50) than in group B (44.0 ± 1.6 years, 41–48), although there was no statistical difference (*P* = NS). MEN1-related aggressive tumours or other malignancies were more frequent in participants with the *CDKN1B* genetic variant (8/24 patients in group A *versus* 5/31 in group B; 33.3 *versus* 16.1%), although this test did not result in statistical significance (*P* = NS) (Table3). The time interval between the date of birth and age of the first aggressive tumour was significantly shorter in group A (median 46 years) than in group B (median not reached; *P* = 0.03). Similarly, the time interval between MEN1 diagnosis and age of the first aggressive tumour was significantly shorter in group A (median 36 months) than in group B (median not reached; *P* = 0.02). Overall survival could not be evaluated as 53/55 patients (96%) were still alive at the time of the study.

**Table 3:** Patients with MEN1-related aggressive tumours and other malignant tumours

Family	Patient no.	*CDKN1B*	Aggressive NET	Other malignancies
1	1	POL	Pancreas >2 cm + LN, L metastases	–
1	2	POL	Pancreas	–
1	3	WT	Pancreas >2 cm + LN metastases	–
2	8	POL	Pancreas >2 cm	–
4	11	POL	Thymus	–
4	14	WT	Pancreas >2 cm	–
7	26	POL	Pancreas >2 cm	–
9	32	POL	Bronchi	–
13	41	WT	Pancreas >2 cm	–
14	43	WT	Pancreas >2 cm	–
16	48	WT	Pancreas >2 cm	–
18	53	POL	Pancreas >2 cm	Renal carcinoma
19	54	POL	Pancreas >2 cm	–

LN: lymph node; L: liver.

## Discussion

The preferred approach to search for low-penetrance cancer susceptibility genes, especially ‘driver’ genes, involves genetic association studies that compare the frequencies of common variants between cases and unaffected controls. The identification of such genes that can be used as prognostic markers is particularly compelling for tumours that do not have a clear genotype/phenotype correlation. MEN1 is a rare syndrome, with inter- and intra-familial variability, without a known genotype–phenotype correlation. Novel genetic prognostic markers that could help in a better management and in predicting the outcome of this syndrome are thus largely needed.

**Figure 1:**
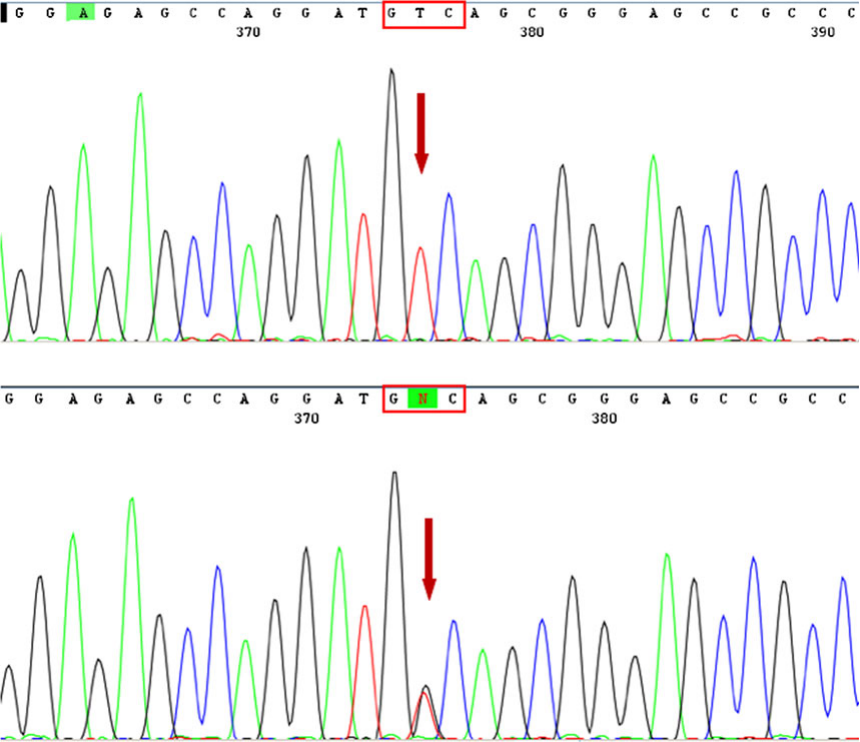
Identification of the CDKN1B V109G single nucleotide polymorphism. Direct sequencing of CDKN1B-amplified exon 1 from two patients’ DNA shows either the wild-type sequence or the T/G transversion at nucleotide 326. The arrow in the lower electropherogram denotes two overlapping peaks, indicative of the presence of two different nucleotides at that position of the DNA sequence. The change causes a valine (GTC) for glycine (GGC) substitution at position 109 of the mature protein.

In this study, we investigated the possible association of *MEN1* malignant tumours with *CDKN1B* V109G polymorphism. Indeed, functional polymorphisms in this gene contribute to the susceptibility and severity of different types of cancers 26,27; in particular, the V109G SNP has extensively been associated with the risk of cancer although inconsistent results have been reported.

Patients selected for this study had all a clinical diagnosis of MEN1, according to the published guidelines 2. The polymorphism in MEN1 patients was as frequent as other types of tumours, whereas it was significantly different from healthy controls. No significant differences were found between the polymorphism and wild-type allele carrying groups according to frequency of MEN1 manifestations as well as diagnostic circumstances.

As previously reported, it should be noted that the genetic screening is helpful to anticipate the identification of MEN1 participants so detecting MEN1-related tumours at an early stage 28,29. This aspect impacts on patients’ prognosis and quality of life but it does not influence the proportion of aggressive tumours that are equally distributed between participants with initial clinical diagnosis and those identified at the genetic screening. The aggressiveness of the tumour seems to be rather an intrinsic characteristic of the tumour. The CDK*N1B* V109G polymorphism could in part explain this characteristic, as it positively correlates with the onset of aggressive tumours. In particular, polymorphism-positive patients displayed a shorter time interval before the occurrence of an aggressive tumour than those negative.

Finally, the polymorphism was associated with specific *MEN1* mutations [c.502G>A, p.G168R in exon 3; c.673T>A, p.W225R in exon 4; c.825+1G>A in intron 5] and patients carrying both genetic variants had a worse prognosis, providing additional support to the association with a more aggressive disease course. Based on these data, it is tempting to speculate that a mutated menin could have an impaired transactivation potential on the target gene *CDKN1B*, as reported 7–10, reducing the amount of the p27/Kip1 produced. Moreover, the V109G polymorphism has been correlated with low levels of p27/Kip1 as it is located in a protein region that physically interacts with Jun activation domain-binding protein 1, an event that could enhance nuclear export and p27/Kip1 degradation 30,31. Disrupting the binding between these two proteins would affect the p27/Kip1 fate and hence cell growth. Thus, the simultaneous presence of specific *MEN1* mutations and the *CDKN1B* polymorphism could potentiate the negative effects of a mutated menin, further reducing p27/Kip1 and its regulatory function. This reasoning could explain at least in part the association found in this tumour syndrome.

In conclusion, we provide the first evidence that among patients who develop *MEN1*-related tumours, those bearing the *CDKN1B* V109G polymorphism have a higher frequency to develop aggressive tumours and hence a more aggressive behaviour, emphasizing the added value of this variant. Thus, the polymorphism may play a role in dictating the evolution of the malignancy whereby *MEN1* acts as a ‘driver’ gene and the *CDKN1B* polymorphism as a ‘modifier’ of tumour progression. While further studies are needed to better elucidate the mechanisms underlying the interplay of this polymorphism with the *MEN1*-gene pathways, we suggest that the association of the polymorphism with a severe prognosis might be used as a novel and early prognostic marker to direct more selective and targeted therapies.
